# Response to Ahlbom *et al.* Comments on Hardell and Carlberg Increasing Rates of Brian Tumors in the Swedish National Inpatient Register and the Causes of Death Register. *Int. J. Environ. Res. Public Health* 2015, *12*, 3793–3813

**DOI:** 10.3390/ijerph120911665

**Published:** 2015-09-17

**Authors:** Lennart Hardell, Michael Carlberg

**Affiliations:** Department of Oncology, Faculty of Medicine and Health, Örebro University, SE-701 82 Örebro, Sweden; E-Mail: michael.carlberg@regionorebrolan.se

We thank Ahlbom *et al*., the authors of [1] for their interest in our paper [2]. Since this is an important issue, the letter deserves a comprehensive and thorough response. Some authors use brain tumor incidence data from Sweden to dismiss evidence of an increased risk for brain tumors associated with the use of mobile and cordless (wireless) phones as published in peer-reviewed scientific literature.

## Incidence/Rate of Brain Tumors

We note that Ahlbom *et al.* have not challenged our main conclusion regarding D43 in the Swedish National Inpatient Register (IPR) [2]: “The main finding in this study was increasing rate of brain tumor of unknown type in the central nervous system (D43)”. Furthermore, we stated that: “In summary this study shows that the Swedish Cancer Register is not reliable to be used to dismiss results in epidemiological studies on the use of wireless phones and brain tumor risk and should not be used as reference for such statements.”

In their letter, Ahlbom *et al.* cite Barlow *et al.* [3]. In fact two of the authors, Holmberg and Talbäck, were co-authoring the study published in 2009 on the completeness of the Swedish Cancer Register. They stated in the article that: “Our study points to amendable problems in reporting. We are aware of a misunderstanding among some clinicians that only cases where the cancer diagnosis has been proven by histology or cytology should be reported, thus they neglect to report cancers diagnosed only clinically. This problem seems to be greater for patients with advanced disease and in a deteriorated condition where no specific anti-tumor therapy is undertaken”.

Their present interpretation of Barlow *et al.* [3] is not correct. The article is not about reporting in the same year, rather they look into a time period: “We selected all malignant cancer cases (according to ICD-10: C00-C96, except C77-C79) in the Hospital Discharge Register from 1998, irrespective of whether the cancer diagnoses were main or secondary…. Use of the patient’s unique personal identification number, a number assigned to each Swedish resident, permitted linkage of individuals to the Swedish Cancer Register for the period 1958–1999, thus giving the hospitals an additional year to report the case to the cancer registry”.

Hence, there were cases that were discharged with a nervous system tumor but were never reported in the preceding several years. Nervous system tumors were among those with the largest underreporting among all solid tumors. Unfortunately, no such study is available for more recent years.

In fact the underreporting might have increased. We discuss that at length in our article, such as the declining frequency of autopsy-based diagnoses, increasing use of CT/MRI for diagnosis, and regional differences in reporting. These facts are not at all discussed by Ahlbom *et al.* in their letter.

Based on our publication [2], Mr. Robert James Parsons, Journaliste indépendant, sent an e-mail on 21 April 2015 to the responsible officer at the Swedish Government regarding the incompleteness of the Swedish Cancer Register. In the reply it was stated that: “The findings in the article are consistent with a previous study conducted by colleagues at the board (Barlow *et al.* 2009 [3]) which is published in a peer-reviewed scientific journal…..This study is also cited each year in the report Cancerincidens i Sverige (Cancer incidence in Sweden), which is the official statistical product of cancer. The problem is therefore considered to be well known by both authorities and researchers who want to use data from the Swedish Cancer Registry. It is incumbent on those who want to use data from the Swedish Cancer Registry to determine if the information is usable for the planned research.” (Letter from Mr. Martin Jeppsson, Public Health and Health Care Division, Ministry of Health and Social Affairs, Government Offices of Sweden, SE-103 33 Stockholm, dated 28 April 2015).

## Causes of Death

Ahlbom *et al.* cite the Causes of Death Register (CDR) in 2010 [4]. In the register comparison is made between 2010 and 2009. The register describes that, for the whole group D37–D48 (Tumors of uncertain or unknown type), an increase of 23% was seen. The number of requests for more specified type of tumor had declined. However, Ahlbom *et al.* omit to cite the conclusion in the report: “It should also be pointed out that the whole change cannot be explained by new work routines but changes in the pattern of causes of death also has an impact.” [4]. We analyzed causes of death in CDR for the time period 1998–2013 and found a joinpoint in 2008, but not between 2009 and 2010. Furthermore, no data are given for tumor of unknown type in the brain or CNS (D43) only, which would be the relevant discussion here.

In contrast to what Ahlbom *et al*. claim, we discuss these items in our article. We publish an increasing rate of D43 in CDR, a change that is not fully explained by the results for malignant brain tumors (C71). The reporting policy has changed, but also highly malignant tumors seem now to be found in the D43 category to a higher extent, which is indicated by the sharply increasing trend of cases of death of this type. These two aspects together lead to the observation that malignant brain tumors have not declined in Sweden.

Furthermore, though not at all mentioned by Ahlbom *et al.*, changes in treatment options with longer survival may influence the results, e.g., a combination of interferon and temozolomide for treatment of glioblastoma [5].

## Mechanistic Aspects

It is pertinent to add some mechanistic aspects that further strengthen the findings of increased brain tumor risk and rate associated with use of wireless phones. These issues were also not considered by Ahlbom *et al.* and would be essential as supplementary evidence on the carcinogenicity of radiofrequency electromagnetic fields (RF-EMFs).

### Reactive Oxygen Species

RF-EMFs do not cause direct DNA damage. On the contrary, numerous studies have shown generation of reactive oxygen species (ROS) that can cause oxidative damage of DNA. This is a well-known mechanism in carcinogenesis for many agents. The broad biological potential of ROS and other free radicals makes radiofrequency radiation a potentially hazardous factor for human health, not only cancer risk but also other health effects [6].

### Tumor Promotion

Tumor promotion by RF-EMF exposure was reported in 2010 in a study on mice [7]. These findings were recently replicated and add to the relevance of tumor risk [8]. Note that Figure 7 in our publication [2] indicates both an early effect in glioma genesis (initiation) and a late effect in carcinogenesis (promotion/progression). This notion is also supported by the results shown in Figures 5 and 6 in our publication [2]. We illustrate this with the number of out-going mobile phone minutes in millions during 1999–2013 with the rates of tumor of unknown type in the brain or CNS (D43) in the Swedish National Inpatient Register and the Causes of Death Register, respectively.

### p53

The p53 protein is a transcription factor that plays a vital role in regulating cell growth, DNA repair and apoptosis, and p53 mutations are involved in disease progression. In a recent study it was found that use of mobile phones for ≥3 hours a day was associated with increased risk for the mutant type of p53 gene expression in the peripheral zone of astrocytoma grade IV (glioblastoma multiforme), and that this increase was statistically significant, correlated with shorter overall survival time [9].

These results are in agreement with the decreased survival for patients with astrocytoma grade IV (glioblastoma multiforme) associated with long-term use of mobile phones and cordless phones that we have reported [10].

## Concluding Remarks

Our article [2] is based on official statistics, and the letter by Ahlbom *et al.* [1] does not necessitate any change in the interpretation of the results. Data on brain tumor incidence in the Swedish Cancer Register should not be used to dismiss an association between use of mobile and cordless phones and brain tumors.

## References

[1] Ahlbom A., Feychting M., Holmberg L., Johansson L.A., Mathiesen T., Pettersson D., SchüZ J., Talbäck M. (2015). Comments on Hardell and Carlberg Increasing Rates of Brian Tumours in the Swedish National Inpatient Register and the Causes of Death Register. Int. J. Environ. Res. Public Health 2015, 12, 3793–3813. Int. J. Environ. Res. Public Health.

[2] Hardell L., Carlberg M. (2015). Increasing rates of brain tumours in the Swedish national inpatient register and the causes of death register. Int. J. Environ. Res. Public Health.

[3] Barlow L., Westergren K., Holmberg L., Talbäck M. (2009). The completeness of the Swedish Cancer Register: A sample survey for year 1998. Acta Oncol..

[4] (2011). Causes of Death 2010. Official Statistics of Sweden. http://www.socialstyrelsen.se/Lists/Artikelkatalog/Attachments/18394/2011-7-6.pdf.

[5] Shen D., Guo C.C., Wang J., Qiu Z.K., Sai K., Yang Q.Y., Chen Y.S., Chen F.R., Wang J., Panasci L., Chen Z.P. (2015). Interferon-α/β enhances temozolomide activity against MGMT-positive glioma stem-like cells. Oncol. Rep..

[6] Yakymenko I., Tsybulin O., Sidorik E., Henshel D., Kyrylenko O., Kyrylenko S. (2015). Oxidative mechanisms of biological activity of low-intensity radiofrequency radiation. Electromagn. Biol. Med..

[7] Tillmann T., Ernst H., Streckert J., Zhou Y., Taugner F., Hansen V., Dasenbrock C.T. (2010). Indication of cocarcinogenic potential of chronic UMTS-modulated radiofrequency exposure in an ethylnitrosourea mouse model. Int. J. Radiat. Biol..

[8] Lerchl A., Klose M., Grote K., Wilhelm A.F., Spathmann O., Fiedler T., Streckert J., Hansen V., Clemens M. (2015). Tumor promotion by exposure to radiofrequency electromagnetic fields below exposure limits for humans. Biochem. Biophys. Res. Commun..

[9] Akhavan-Sigari R., Baf M.M., Ariabod V., Rohde V., Rahighi S. (2014). Connection between cell phone use, p53 gene expression in different zones of glioblastoma multiforme and survival prognoses. Rare Tumors.

[10] Carlberg M., Hardell L. (2014). Decreased survival of glioma patients with astrocytoma grade IV (glioblastoma multiforme) associated with long-term use of mobile and cordless phones. Int. J. Environ. Res. Public Health.

